# Pool Chemical Injuries in Public and Residential Settings — United States, 2008–2017, and New York, 2018

**DOI:** 10.15585/mmwr.mm6819a2

**Published:** 2019-05-17

**Authors:** Kayla L. Vanden Esschert, Tadesse Haileyesus, Amanda L. Tarrier, Michelle A. Donovan, Gary T. Garofalo, Joseph P. Laco, Vincent R. Hill, Michele C. Hlavsa

**Affiliations:** ^1^Division of Foodborne, Waterborne, and Environmental Diseases, National Center for Emerging and Zoonotic Infectious Diseases, CDC; ^2^Oak Ridge Institute for Science and Education, Oak Ridge, Tennessee; ^3^Division of Analysis, Research and Practice Integration, National Center for Injury Prevention and Control, CDC; ^4^New York State Department of Health; ^5^Hornell District Office, New York State Department of Health; ^6^Division of Environmental Health Science and Practice, National Center for Environmental Health, CDC.

Pool chemicals are added to water in treated recreational water venues (e.g., pools, hot tubs/spas, and water playgrounds) primarily to protect public health. Pool chemicals inactivate pathogens (e.g., chlorine or bromine), optimize pH (e.g., muriatic acid), and increase water clarity, which helps prevent drowning by enabling detection of distressed swimmers underwater. However, pool chemicals can cause injuries if mishandled. To estimate the annual number of U.S. emergency department (ED) visits for pool chemical injuries, CDC analyzed 2008–2017 data from the National Electronic Injury Surveillance System (NEISS), operated by the U.S. Consumer Product Safety Commission (CPSC). During 2015–2017, pool chemical injuries led to an estimated 13,508 (95% confidence interval [CI] = 9,087–17,929) U.S. ED visits; 36.4% (estimated 4,917 [95% CI = 3,022–6,811]) of patients were aged <18 years. At least 56.3% (estimated 7,601 [95% CI = 4,587–10,615]) of injuries occurred at a residence. Two thirds of the injuries occurred during the period from Memorial Day weekend through Labor Day. This report also describes a toxic chlorine gas incident that occurred at a public pool in New York in 2018. Pool chemical injuries are preventable. CDC’s Model Aquatic Health Code (MAHC) is an important resource that operators of public treated recreational water venues (e.g., at hotels, apartment complexes, and waterparks) can use to prevent pool chemical injuries.

NEISS captures data on ED visits for injuries, including those associated with consumer products. NEISS records include data on consumer products (swimming pool chemical product code = 938); patient age, sex, and race/ethnicity; the most severe diagnosis; the most seriously injured body part; patient disposition; incident location; and two 71-character narrative fields to describe the incident leading to injury. These data are collected from a nationally representative probability sample of approximately 100 hospitals across the United States, and thus, can be used to calculate national estimates. Each case was weighted based on the inverse probability of hospital selection, and the weights were summed to produce national estimates; 95% CIs were calculated according to CPSC’s direct variance method, accounting for the complex sampling design (*1*). Rates per 100,000 population were calculated using weighted NEISS point estimates and U.S. Census Bureau population estimates (*2*). Descriptive analyses of 2015–2017 data were conducted to characterize the most recent pool chemical injuries and increase national estimate stability. Data analyses were conducted using SAS (version 9.4; SAS Institute).

During 2008–2017, the median estimated annual number of U.S. ED visits for pool chemical injuries was 4,535 (range = 3,151–5,215) (Figure). During 2015–2017, pool chemical injuries led to an estimated 13,508 total ED visits (95% CI = 9,087–17,929; rate = 1.4 per 100,000 population) (Table), with persons aged <18 years accounting for 36.4% of patients (estimated 4,917 [95% CI = 3,022–6,811]). An estimated 93.9% (95% CI = 8,480–16,899) of patients seeking care in an ED for pool chemical injuries were either treated in the ED and released or examined in the ED and released without treatment. An estimated 5,245 patients (95% CI = 3,135–7,355; rate 0.5 per 100,000 population) had their injury diagnosed as poisoning. NEISS report narratives indicated that approximately 90% of patients who received a diagnosis of poisoning were injured via inhalation rather than ingestion. The poisoning diagnosis contributed to “all parts of the body (>50% of the body)”* being the most affected body part. An estimated 3,745 injuries (95% CI = 2,497–4,994) were diagnosed as dermatitis or conjunctivitis, and an estimated 2,588 (95% CI = 644–4,533]) were diagnosed as chemical burn. No deaths were documented. At least 56.3% (estimated 7,601 [95% CI = 4,587–10,615]) of injuries occurred at a residence. Among the estimated 9,065 injuries for which incident location data were captured, 83.8% (7,601 [95% CI = 4,587–10,615]) occurred at a residence. Approximately two thirds (64.5%) of all ED visits occurred during the summer swim season (Saturday of Memorial Day weekend [late May] through Labor Day [first Monday in September]). Narratives for NEISS reports noted that patients were most frequently injured when inhaling chemical fumes or dust (particularly while opening containers), when pool chemicals were not secured away from children, or when pool chemicals were added to the water just before the patient entered the water.

**FIGURE:**
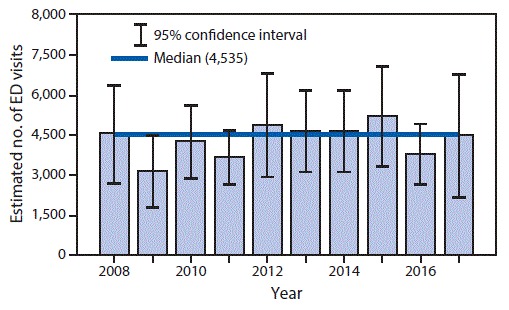
Estimated number of emergency department (ED) visits for pool chemical injuries, by year — National Electronic Injury Surveillance System, United States, 2008–2017

**TABLE:** Estimated number, percentage, and rate of pool chemical injuries treated in emergency departments — National Electronic Injury Surveillance System (NEISS), United States, 2015–2017

Characteristic	Sample case count	Weighted estimate* (95% CI)	%	Annual rate^†^
**Total**	**298**	**13,508 (9,087–17,929)**	**100.0**	**1.4**
**Patient age group (yrs)**				
0–17	130	4,917 (3,022–6,811)	36.4	2.2
18–45	83	3,977 (2,505–5,448)	29.4	1.1
46–64	55	3,036 (1,855–4,218)	22.5	1.3
≥65	30	1,579 (774–2,383)	11.7	1.1
**Patient sex**				
Male	181	8,735 (5,372–12,098)	64.7	1.8
Female	117	4,773 (3,369–6,177)	35.3	1.0
**Patient race/ethnicity**				
White, non-Hispanic	127	6,545 (4,189–8,902)	48.5	–
Black, non-Hispanic	25	1,008 (301–1,715)	7.5	–
Hispanic	20	572 (107–1,036)	4.2	–
Other (e.g., multiple race)	11	141 (0–343)	1.0	–
Unknown	115	5,242 (822–9,663)	38.8	–
**Injury diagnosis**				
Poisoning^§^	117	5,245 (3,135–7,355)	38.8	0.5
Dermatitis or conjunctivitis	85	3,745 (2,497–4,994)	27.7	0.4
Chemical burns	49	2,588 (644–4,533)	19.2	–
Other or not stated^¶^	47	1,930 (1,187–2,673)	14.3	0.2
**Affected body part**				
All parts of the body (>50% of the body)**	140	6,371 (4,117–8,624)	47.2	0.7
Eye	96	4,451 (2,561–6,342)	33.0	0.5
Other^††^	62	2,686 (1,726–3,646)	19.9	0.3
**Patient disposition**				
Treated and released or examined and released without treatment	270	12,690 (8,480–16,899)	93.9	1.3
Treated and admitted for hospitalization (within same facility)	16	593 (186–1,001)	4.4	–
Other^§§^	12	225 (24–426)	1.7	–
**Incident location**				
Residence	144	7,601 (4,587–10,615)	56.3	–
Place of recreation or sports	50	891 (285–1,497)	6.6	–
Other public property^¶¶^	15	573 (202–945)	4.2	–
Unknown	89	4,443 (2,458–6,428)	32.9	–

**Abbreviation:** CI = confidence interval.

* Estimates might not sum to total because of rounding.

^†^ Rates per 100,000 population. If the sample count was <20, the estimate <1,200, or the coefficient of variation >30%, then the rate was potentially unstable and not reported. Rates by incident location and race/ethnicity are not reported because of the high percentage of missing data.

^§^ Poisoning includes inhalation of vapors, fumes, or gases, as well as ingestion.

^¶^ Other diagnoses (sample case counts): other or not stated (N = 26), contusions or abrasions (six), submersion (three), fracture (two), laceration (two), anoxia (two), radiation (two), internal organ injury (one), foreign body (one), aspiration (one), and thermal burn (one).

** For a poisoning injury diagnosis, NEISS requires that affected body part be coded as “all parts of the body (>50% of the body).”

^††^ Other most commonly injured body parts (sample case counts): face (10), upper trunk (nine), hand (nine), lower arm (eight), and other (26).

^§§^ Other dispositions (sample case counts): left without being seen (seven), held for observation (four), and transferred to another hospital (one).

^¶¶^ NEISS defines “other public property” as hotels/motels, stores, office buildings, etc.

New York mandates operators of public treated recreational water venues to report onsite illness or injury incidents^†^ to permitting officials within 24 hours of occurrence (https://www.health.ny.gov/regulations/nycrr/title_10/part_6/subpart_6-1.htm). In August 2018, maintenance personnel at an outdoor pool in upstate New York noticed a yellow substance seeping into the pool through the inlets (e.g., water jets). Lifeguards cleared the pool of swimmers, and the maintenance personnel examined the equipment room. There they discovered that the recirculation pump was not running, resulting in no water flow in the recirculation system.^§^ The operator turned the pump back on, which resulted in resumption of water flow in the recirculation system. Consequently, substantially more of the yellow substance entered the pool; a pungent odor developed; and lifeguards evacuated the pool area. Investigation of the event suggested that a power outage in the area the previous night could have shut down the recirculation pump; however, the water flow monitoring system, which deactivates the chemical feeders when there is no water flow, failed. The failure to automatically shut off the chemical feeders allowed concentrated chlorine and acid to mix, and thus, generated toxic chlorine gas in the recirculation system. Persons in the pool area reported blisters, nausea, vomiting, or irritation of the face or eyes, and a few followed up with a health care provider.

## Discussion

Maximizing the health benefits of water-based physical activity (*3*) includes minimizing the risk for pool chemical injuries (*4*–*8*) and transmission of pathogens (*9*). The magnitude of U.S. ED visits for pool chemical injuries, the disproportionate impact on children, and the incidence of these injuries at residences all call for increased awareness about pool chemical safety among operators of public venues and owners of residential venues.

Recommendations to minimize risk for illness and injury associated with public treated recreational water venues can be found in the MAHC (https://www.cdc.gov/mahc). The MAHC is based on the latest science or best practices and can be adopted voluntarily, in part or whole, by state and local jurisdictions. For example, the MAHC recommends including pool chemical safety in training for operators of public pools, hot tubs/spas, and water playgrounds (MAHC 6.1.2.1.4.6^¶^), covering topics such as how to read product labels. Labels include information on what chemicals are incompatible (e.g., chlorine and acid) and which personal protective equipment to use. The MAHC also recommends automatic deactivation of chemical feeders in the event of no or low water flow in the recirculation system (MAHC 4.7.3.2.1.3). In the upstate New York incident, the water flow monitoring system installed to do this (as mandated by the state code) failed. The pool remained closed for the duration of the 2018 summer swim season, and the flow indicator was replaced. In coordination with the New York State Department of Health, new policies and procedures will be implemented at the venue in the 2019 season, including performing a check of the recirculation system, testing pool water chemistry, and documenting findings every 2 hours while the pool is open to swimmers. The next edition of the MAHC is scheduled for update and release in 2021. CDC asks state and local public health officials, who are on the frontline of investigating and preventing recreational water–associated illness and injury, to take an active role in the MAHC updating process. This starts with submitting MAHC change requests based on findings from investigations and implementation of prevention measures. To be considered for the 2021 MAHC (4th edition), MAHC change requests can be submitted to the Council for the MAHC (CMAHC; https://www.cmahc.org/enter-change-request.php) by January 6, 2020.

The findings in this report are subject to at least five limitations. First, although NEISS data provide a snapshot of pool chemical injuries leading to ED visits, they do not characterize the epidemiology of pool chemical injuries that do not result in an ED visit. Second, understanding of pool chemical injuries is limited by minimal data (e.g., restricted text fields that preclude detailed description of incidents leading to injury) and missing data (e.g., incident location). Third, because NEISS collects data on only the most severe diagnosis, some pool chemical injuries might have been missed. Fourth, in some injury reports, the injury-causing chemical could have been incorrectly identified. For example, the disinfection byproduct, chloramine (chlorine combined with nitrogenous compounds such as those found in urine, feces, sweat, and dirt) might have been the cause of ocular irritation rather than chlorine itself as was reported. Finally, water chemistry can change quickly, making it difficult to determine the etiology of and factors contributing to pool chemical injuries.

State and local jurisdiction over residential treated recreational water venues is limited compared with jurisdiction over public venues. However, to help prevent pool chemical injuries in the residential setting, state and local environmental health practitioners can be a resource for residential pool or hot tub/spa owners by offering them pool chemical safety training. Pool chemical safety recommendations (https://www.cdc.gov/healthywater/swimming/aquatics-professionals/preventing-pool-chemical-events.html) are generally the same for residential and public venues (Box). Swimmers and parents of young swimmers should understand basic water chemistry and how they can help optimize it before getting into a pool, hot tub/spa, or water playground. To help prevent the formation of chloramines, which cause ocular and respiratory irritation and consume chlorine that would otherwise be available to inactivate pathogens, swimmers should take a rinse shower before getting in the water, not urinate or defecate in the water, and take children on bathroom breaks or check diapers every hour. These steps help limit the amount of nitrogen compounds being introduced into the water. Healthy and Safe Swimming Week (the week before Memorial Day) is an ideal time to disseminate these messages.

BOX CDC recommendations to prevent pool chemical injuries*

**Before using pool chemicals**
  - Get trained in pool chemical safety (for example, during operator training course)
  - Ask for help if you are not trained for specific tasks
  - Read entire product label or safety data sheet (SDS) before using
  - Learn your pool’s Emergency Chemical Spill Response Plan and practice steps (e.g., evacuation)
**Using pool chemicals safely**
  - Dress for safety by wearing appropriate safety equipment (e.g., safety goggles, gloves, and mask)
  - Read chemical product label before each use
    - Handle in a well-ventilated area
    - Open one product container at a time and close it before opening another
    - Minimize dust, fumes, and splashes
    - Measure carefully
    - Never mix chlorine products with acid; this could create toxic gases
    - Never mix different pool chemicals (e.g., different types of chlorine products) with each other or with any other substance
  - Only predissolve pool chemicals when directed by product label
    - If product label directs predissolving, add pool chemical to water; never add water to pool chemical because violent (potentially explosive) reaction can occur

* To order free laminated pool chemical safety posters (one on safe storage and one on safe use), go to https://wwwn.cdc.gov/pubs/CDCInfoOnDemand.aspx?ProgramID=93.

**Summary**

**What is already known about this topic?**

Pool chemicals are added to water in treated recreational water venues (e.g., pools, hot tubs/spas, and water playgrounds) to prevent illnesses and outbreaks; these same chemicals can cause injuries if mishandled.

**What is added by this report?**

During 2015–2017, pool chemical injuries led to an estimated 13,508 U.S. emergency department visits, approximately one third of which occurred in persons aged <18 years. Most injuries occurred at a residence, and two thirds occurred during the summer swimming season (Memorial Day weekend through Labor Day).

**What are the implications for public health practice?**

Pool chemical injuries are preventable. CDC’s Model Aquatic Health Code (https://www.cdc.gov/mahc), based on the latest science or best practices, is an important resource to prevent pool chemical injuries.

## Footnotes

* For a poisoning injury diagnosis, NEISS requires that affected body part be coded as “all parts of the body (>50% of the body).”

† This includes incidents that result in death, require resuscitation, or require referral to a hospital or other facility for medical attention or where illness is associated with bathing water quality.

§ The recirculation system transports water from a treated recreational water venue via outlets (e.g., the main drain). Via the venue plumbing, the water passes through the filter. Subsequently, chemical feeders add chlorine or bromine and further along in the plumbing, a pH-adjusting chemical (e.g., muriatic acid). This means chemical dilution occurs before mixing. Finally, the recirculation system returns treated water into the venue via inlets.

¶ For reference purposes, MAHC elements discussed in this report are followed by the specific section number that covers that element.

## References

[1] Consumer Product Safety Commission. The NEISS sample (design and implementation) 1997 to present; Bethesda, MD: Consumer Product Safety Commission; 2019. https://www.cpsc.gov/s3fs-public/pdfs/blk_media_2001d011-6b6.pdf

[2] US Census Bureau. Annual estimates of the resident population by single year of age and sex for the United States: April 1, 2010 to July 1, 2017; 2017 population estimates. Washington, DC: US Department of Commerce, US Census Bureau; 2019. https://factfinder.census.gov/faces/tableservices/jsf/pages/productview.xhtml?pid=PEP_2017_PEPSYASEXN&prodType=table

[3] CDC. Health benefits of water-based exercise. Atlanta, GA: US Department of Health and Human Services, CDC; 2019. https://www.cdc.gov/healthywater/swimming/swimmers/health_benefits_water_exercise.html

[4] Wilken JA, DiMaggio M, Kaufmann M, Inhalational chlorine injuries at public aquatic venues—California, 2008–2015. MMWR Morb Mortal Wkly Rep 2017;66:498–501. 10.15585/mmwr.mm6619a3 28520711 PMC5657649

[5] Hlavsa MC, Robinson TJ, Collier SA, Beach MJ. Pool chemical–associated health events in public and residential settings—United States, 2003–2012, and Minnesota, 2013. MMWR Morb Mortal Wkly Rep 2014;63:427–30. 24827410 PMC5779411

[6] Anderson AR, Welles WL, Drew J, Orr MF. The distribution and public health consequences of releases of chemicals intended for pool use in 17 states, 2001–2009. J Environ Health 2014;76:10–5. 24909007

[7] CDC. Acute illness and injury from swimming pool disinfectants and other chemicals—United States, 2002–2008. MMWR Morb Mortal Wkly Rep 2011;60:1343–7. 21976116

[8] CDC. Pool chemical–associated health events in public and residential settings—United States, 1983–2007. MMWR Morb Mortal Wkly Rep 2009;58:489–93. 19444152

[9] Hlavsa MC, Cikesh BL, Roberts VA, Outbreaks associated with treated recreational water—United States, 2000–2014. MMWR Morb Mortal Wkly Rep 2018;67:547–51. 10.15585/mmwr.mm6719a3 29771872 PMC6048947

